# The use of repetitive transcranial magnetic stimulation for treatment of obsessive-compulsive disorder: a scoping review

**DOI:** 10.1108/MIJ-05-2021-0002

**Published:** 2021-09-17

**Authors:** Medard Kofi Adu, Ejemai Eboreime, Adegboyega Oyekunbi Sapara, Andrew James Greenshaw, Pierre Chue, Vincent Israel Opoku Agyapong

**Affiliations:** Department of Psychiatry, Faculty of Medicine and Dentistry, University of Alberta Edmonton Canada; Department of Psychiatry, Faculty of Medicine and Dentistry, University of Alberta Edmonton Canada; Department of Psychiatry, Faculty of Medicine and Dentistry, University of Alberta Edmonton Canada; Department of Psychiatry, Faculty of Medicine and Dentistry, University of Alberta Edmonton Canada; Department of Psychiatry, Faculty of Medicine and Dentistry, University of Alberta Edmonton Canada; Department of Psychiatry, Faculty of Medicine and Dentistry, University of Alberta Edmonton Canada

**Keywords:** Post-traumatic stress disorder, Bipolar disorders, Repetitive transcranial magnetic stimulations, Treatment, Obsessive-compulsive disorder

## Abstract

**Purpose:**

This paper aims to explore the relevant literature available regarding the use of repetitive transcranial magnetic stimulation (rTMS) as a mode of treatment for obsessive-compulsive disorder (OCD); to evaluate the evidence to support the use of rTMS as a treatment option for OCD.

**Design/methodology/approach:**

The authors electronically conducted data search in five research databases (MEDLINE, CINAHL, Psych INFO, SCOPUS and EMBASE) using all identified keywords and index terms across all the databases to identify empirical studies and randomized controlled trials. The authors included articles published with randomized control designs, which aimed at the treatment of OCD with rTMS. Only full-text published articles written in English were reviewed. Review articles on treatment for conditions other than OCD were excluded. The Covidence software was used to manage and streamline the review.

**Findings:**

Despite the inconsistencies in the published literature, the application of rTMS over the supplementary motor area and the orbitofrontal cortex has proven to be promising in efficacy and tolerability compared with other target regions such as the prefrontal cortex for the treatment of OCD. Despite the diversity in terms of the outcomes and clinical variability of the studies under review, rTMS appears to be a promising treatment intervention for OCD.

**Research limitations/implications:**

The authors of this scoping review acknowledge several limitations. First, the search strategy considered only studies published in English and the results are up to date as the last day of the electronic data search of December 10, 2020. Though every effort was made to identify all relevant studies for the purposes of this review per the eligibility criteria, the authors still may have missed some relevant studies, especially those published in other languages.

**Originality/value:**

This review brought to bare the varying literature on the application of rTMS and what is considered gaps in the knowledge in this area in an attempt to evaluate and provide information on the potential therapeutic effects of rTMS for OCD.

## Introduction

Transcranial magnetic stimulation (TMS) is a non-invasive neuromodulatory intervention, which affects neural activity through rapidly alternating magnetic fields. The stimulation operates through Faraday’s law of electromagnetic induction, where the rapidly alternating electric current in the stimulating coil placed over the scalp generates a magnetic field that moves across the skull and produces electric currents in the neural tissue beneath (Wagner *et al.*, 2009). This magnetic field has the capacity to penetrate the bone of the skull to stimulate cortical activity. Pulses can be delivered in a repeated manner to induce long-term changes in neural activity (Dhaliwal *et al.*, 2015) as an increase or a decrease in cortical excitability through relatively high (>5 Hz) or low frequency (1 Hz) stimulation (Rossi *et al.*, 2009; Wassermann *et al.*, 1996). Repetitive transcranial magnetic stimulation (rTMS) is very flexible and, depending on the site and frequency, it can inhibit or induce local and remote brain activity (Liu *et al.*, 2007). Typical rTMS comprises a train of repetitive pulses with similar stimulus intervals (Dhaliwal *et al.*, 2015; Sandrini *et al.*, 2011).

Barker (1985) originally introduced TMS as a safe, and painless non-invasive means of applying focal brain stimulation, to stimulate the motor cortex and to assess human central motor pathways (Barker *et al.*, 1985). rTMS has become an integral research tool in psychiatric treatment as method to exert explicit effects on a range of measures of brain function (Hallett, 2000; Rossini and Rossi, 2007). rTMS has been evaluated quite extensively as a therapeutic tool for several psychiatric disorders and is accepted as a brain-system-based, neuromodulation treatment for impacting direct targets involved in the neural circuitry of these disorders (Nahas *et al.*, 2001).

A previous review of rTMS studies identified limitations in earlier clinical trials and recommended further research (Daskalakis *et al.*, 2008). Results of more recent studies report improved rTMS outcomes through higher or accelerated dosing regimens (Hadley *et al.*, 2011; Holtzheimer *et al.*, 2010), extended treatment durations (McDonald *et al.*, 2011), patient centered stimulation frequencies (Speer *et al.*, 2009) and bilateral stimulation (Blumberger *et al.*, 2012). Further, they define more accurate and advanced neuro-navigational technologies (Fitzgerald *et al.*, 2009) and more precise techniques for detecting the dorsolateral prefrontal cortex (DLPFC) (Herbsman *et al.*, 2009) with newer coil geometries (Levkovitz *et al.*, 2007). With these advancements, new rTMS studies have reported higher scores in remission and response, ranging from 30%–35% and 40%–55%, respectively (Holtzheimer *et al.*, 2010; Galletly *et al.*, 2012; Levkovitz *et al.*, 2009).

Generally, rTMS treatments are comparatively simple and relatively easy to administer, are non-invasive and are typically well-tolerated by patients (Pink *et al.*, 2021). A major benefit of rTMS is its relative safety being devoid of any major adverse side-effects (Machii *et al.*, 2006). It is a highly cost-effective alternative to other more expensive treatment methods such as electroconvulsive therapy (Coles *et al.*, 2018). The most frequent negative effect noticed by patients is temporary pain in the scalp, although with a moderate increase in the intensity of rTMS, it should be normalized (Perera *et al.*, 2016). Vasovagal syncope may also manifest at the initial stages of the treatment and caution is taken to not avoid having the patient stand up, in addition, earplugs can help reduce the clicking sound experienced during rTMS administration (Tringali *et al.*, 2012). rTMS was approved in Canada in 2002 and in the USA in 2008 (Kennedy *et al.*, 2009; Höflich *et al.*, 1993). In 2015, it was also approved by the National Institute for Health and Care Excellence for treatment-resistant depression in the UK (Fregni *et al.*, 2005; Hara *et al.*, 2016).

The large literature on superficial brain stimulation for mental disorders is based on rTMS for major depressive disorder (Cristancho *et al.*, 2013a). Based on its versatility and efficacy, rTMS use has now been investigated in other psychiatric conditions including bipolar disorders, psychotic disorders, anxiety disorders, obsessive-compulsive disorder (OCD) and post traumatic stress disorders (PTSD) (Cristancho *et al.*, 2013b). Evidence-based guidelines for the therapeutic use of rTMS (Lefaucheur *et al.*, 2014) drew attention to the analgesic effect of high frequency (HF) rTMS of the motor cortex and the antidepressant effect of HF rTMS of the DLFPC. Similar encouraging outcomes have been reported for neuropsychiatric conditions such as schizophrenia and motor stroke. It has also been revealed that rTMS is capable of regulating cortical plasticity and brain network movements. The outcome depends on the selected cortical section and the different stimulating parameters such as the frequency, design and the potency of stimulations (Lefaucheur, 2008; Lefaucheur, 2012). Many studies including a meta-analysis confirm the antidepressant effects of rTMS of the DLFPC (Burt *et al.*, 2002; Couturier, 2005), but there seems to be conflicting outcomes in relation to anxiety disorders (Herwig *et al.*, 2007; O’Reardon *et al.*, 2007).

Although antidepressants or psychotherapy help the symptoms of patients with OCD, this condition can be very debilitating and presents with a greater degree of non-response to conventional treatments (Ressler and Mayberg, 2007). Despite the wide use of rTMS for the management of mental disorders and the continuous interest in research for newer treatments for OCD, the therapeutic use of rTMS is still focused in the domain of depression (Schoenfeldt-Lecuona *et al.*, 2010), and much less is known and evaluated for its use in the management of OCD.

In view of the above considerations, the clinical effectiveness of rTMS should be assessed in relation to its potential to provide OCD patients with safe, and lasting improvement in quality of life (Machado *et al.*, 2012; Grant and Booth, 2009). This scoping review aims to identify what we know and to consider gaps in our knowledge in this area in an attempt to evaluate and provide information on the potential therapeutic effects of rTMS for OCD.

## Methods

We developed an operationalized search strategy, which was applied to an electronically conducted data search in five research databases (MEDLINE, CINAHL, Psych INFO, SCOPUS and EMBASE) using relevant keywords and index terms across all the databases to identify empirical studies and randomized controlled trials (RCTs).

Key terms included: rTMS, OCD, Post-traumatic stress disorder, Bipolar disorders and Treatment. This was a larger search strategy involving results for the use of rTMS for the treatment of three major mental disorders (OCD, PTSD and Bipolar Disorders). This paper reports only on and discusses the results specifically for OCD. Table 1 shows a sample of the search strategy, for Medline.

Two independent reviewers (Medard Adu and Ejemai Eboreime) conducted the title and abstract screening, as well as the full text screening and came out with relevant articles that conformed to the objectives of the scoping review. Thematic classifications were done by the first reviewer (MA), with decisions analyzed by the second reviewer (EE). Where conflicts in classification arose, the articles in question were scrutinized and consensus was reached between the two reviewers.

### Inclusion and exclusion criteria

Inclusion criteria included studies involving a completed RCT of rTMS as a treatment intervention for OCD. Open label trials on OCD using rTMS as a treatment intervention were also included. The review only covered full text articles and studies published in English. Studies involving rTMS as a form of treatment for PTSD, Bipolar disorders, OCD with comorbidities or studies involving any other conditions other than OCD, as well as those examining rTMS as a combined therapy with pharmacotherapy or any other interventions were excluded. Systematic reviews, meta-analysis and study protocols and experiments with rTMS that were not designed for treatment for OCD were not included.

## Results

**Figure 1:**
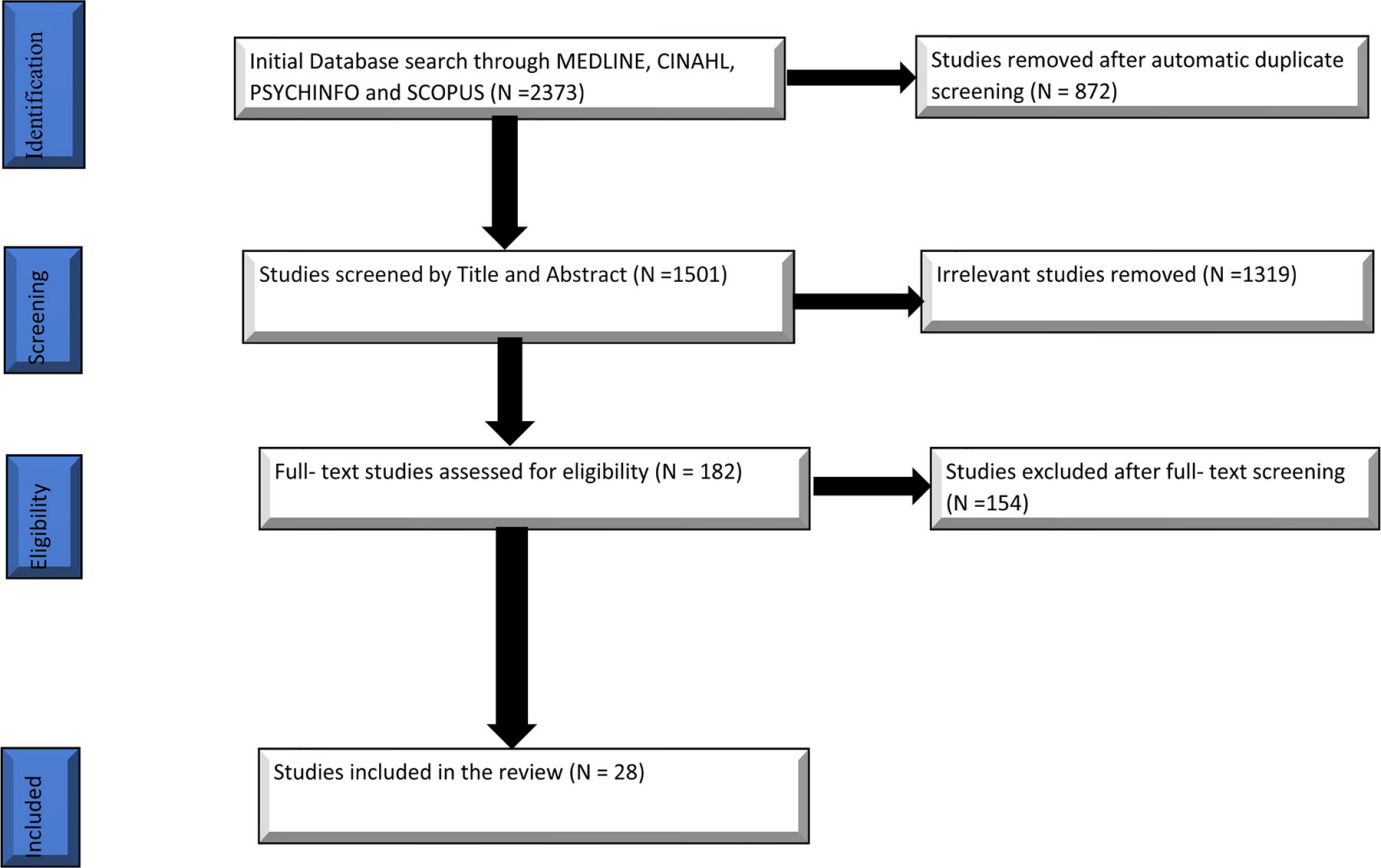
Prisma flow diagram summarizing search process and results

Through the search strategy and the use of the Covidence software, we identified a total of 2,373 studies from the electronic databases searched. The Covidence software automatically screened and removed 872 studies as duplicates. The remaining items (1,501) were screened against the eligibility criteria set by the authors based on the title and abstract only, yielding 182 remaining records for full text screening. In total, 154 studies were excluded in the full text screening phase, leaving a final pool of 28 studies that were eligible for inclusion in this scoping review (Figure 1).

Many of the studies examined rTMS as a stand-alone treatment intervention for OCD with most of them comparing the use and efficacy of rTMS to sham treatment. Relevant and detailed methodological information was extracted and summarized from the various studies and presented in Table 2.

**Figure 2:**
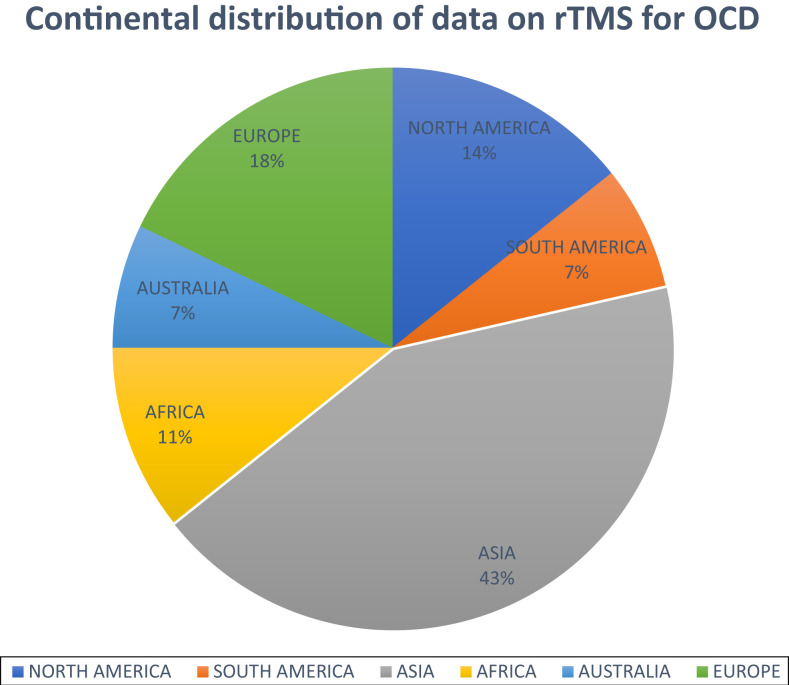
Number of studies extracted from the various continents (*n* = 28)

We examined the geographical distribution of studies conducted on rTMS treatment for OCD globally, as presented in Figure 2. Out of the total of 28 studies included in our review, 12 (43%) were conducted in Asia, North America and South America had 4 (14%) and 2 (7%) studies, respectively, Europe had 5 (18%), Africa had 3 (11%) and Australia had 2 (7%) studies. This indicates that research on rTMS in OCD is being conducted across all continents, but the quantity and scope vary widely across geographical jurisdictions. Table 2 summarizes the main findings for these included studies.

Study designs vary widely, including 18 RCTs, 4 open-label trials, 4 retrospective analysis, 1 brief report and 1 case report. All these studies sought to evaluate the efficacy and effectiveness of rTMS for the treatment of OCD. Sample sizes ranged from 10 to 100 subjects across included studies with a mean sample size of 31.68. The studies were heterogeneous in terms of features of clinical variability such as the severity of OCD symptoms, duration of sickness and rate of resistance to pharmacotherapy. Location of rTMS stimulation, varied among studies, as did treatment duration and stimulus intensity. Of the 28 studies included, 19 used 70 mm figure-of-eight shaped coils because of their ability to induce more focal current compared to circular coils. The remaining studies variously used the 9 cm circular coil, DB-80 butterfly double-cone coil and the H-shaped coil design. Duration of treatment varied across studies, from two weeks to seven weeks. In total, 19 studies applied rTMS with a low frequency and eight applied HF ranging from 10 Hz to 20 Hz, the one remaining study of the 28 compared effects of low and HF treatment protocols.

In total, 19 studies (68%) reported significant positive outcomes and the other 9 studies reported no significant symptom improvement. In each of the included studies, rTMS application was reported as well-tolerated with no significant side-effects, although there were a few reports of mild side-effects such as mild headache, dizziness and scalp pain, across the studies.

## Discussion

The 28 studies under review suggest that rTMS has potential as a safe and clinically efficacious treatment intervention for OCD. Despite the diverse outcome measures included in this selection of studies, there were some consistent significant OCD symptom improvements.

Many factors may have accounted for the varying effectiveness of the application of rTMS across the studies and major domains of outcomes. For instance, rTMS treatment protocols and stimulation parameters vary greatly across studies, with poorly defined intervention protocols. Another factor is that different measuring tools are used to evaluate similar outcomes across studies, making a comparative evaluation of results difficult. It also makes it difficult to understand which rTMS parameters lead to the most significant outcomes and treatment response.

However, due to the diverse nature and presentation of mental conditions, it may seem unrealistic to think uniquely of an optimal or even a standardized rTMS protocol that will work across studies of the different conditions even if they target similar symptoms. One important aspect of rTMS, as identified in this review is its versatility, which allows for the development and adaption of protocols addressing similar symptoms from different conditions with potentially positive outcomes.

### Targeted brain regions of repetitive transcranial magnetic stimulation

The pathophysiology of OCD according to structural and functional neuroimaging studies is linked with the dysfunction of the orbitofronto-striato-pallido-thalamic circuitry, which includes the orbitofrontal cortex (OFC), Dorsolateral prefrontal cortex (DLPFC) and the medial PFC, as well as the thalamus (Saxena and Rauch, 2000; Van Den Heuvel *et al.*, 2005). Modulation of this circuitry by neurosurgical mechanisms and by means of deep brain stimulation has proven to be effective in reducing symptoms of OCD (Mallet *et al.*, 2008). Bearing in mind the possibility of rTMS in modulating cortical and subcortical structures of the brain, the possible therapeutic effects of rTMS have been extensively studied and evaluated in literature in the quest to normalizing hyper- or hypoactive brain regions by targeting dysfunctional cortico- subcortical circuits in people with OCD.

For many of the studies extracted, the locus of rTMS stimulation was at either the left-DLPFC or the right-DLPFC and with high or low frequency rTMS. The overall accepted rationale is that the DLPFC could be a possible starting point for the induction of remote stimulation in the cortico-subcortical circuits connected. For most of the trials, the left- dorsolateral prefrontal cortex (LDLPFC) and right- dorsolateral prefrontal cortex (RDLPFC) were stimulated with the “5 cm method” where the figure-of-eight coil was centered on a point at 5 cm rostral to and in the same sagittal line as the optimal area for activating the right or left abductor pollicis brevis muscles during motor threshold (MT) assessment (Sachdev *et al.*, 2007; Praško *et al.*, 2006; Greenberg *et al.*, 1997). As prefrontal mechanisms are implicated in OCD, Greenberg *et al.* (1997) undertook a non-sham-controlled, single-blind rTMS study on the evidence of PFC hypermetabolism and hyperperfusion in untreated OCD patients. The preliminary results suggest that DLPFC rTMS had modest, lateralized effects on compulsions but not obsessions.

From the data extracted, another brain region studied for the administration of rTMS is the OFC. As indicated earlier, the OFC performs a very important function in the pathophysiology of OCD and because obsessions and compulsions are deemed to be mediated at least in part by the hyperactivity in the orbitofrontal-subcortical circuits and the increase in functional activity in the OFC. Inspired by the fact that OFC rTMS may seem OCD-specific, a randomized, single blind sham-controlled study was conducted by Ruffini *et al.* (2009). The researchers evaluated the efficacy of LF-rTMS over the left OFC with a low frequency (1 Hz) rTMS at 80% RMT for 3 weeks. There was a significant reduction in Yele-Brown obsessive compulsive scale (YBOCS) scores for the active group after the 3^rd^ and 10^th^ weeks compared to sham treatment.

The supplementary motor area (SMA) is one of the most recent brain targets used for the application of rTMS and evidence suggests that the motor and premotor cortex are hyperexcitable in OCD. An open-label trial conducted by Mantovani *et al.* (2006) sought to evaluate whether low-frequency rTMS to the SMA could normalize overactive motor cortical regions and thereby improve symptoms of patients with OCD. There was clinical improvement at the end of the first week of the treatment with rTMS and by the second week, there was a statistically significant improvement in the reductions seen in Yele-Brown obsessive compulsive scale (YBOCS), Clinical Global Impression, Beck depression inventory (BDI), Hamilton depression rating scale (HDRS), Hamilton anxiety rating scale (HARS) and Symptom Checklist-90. Following the publication of this study, many of the most recent trials on rTMS application for the treatment of drug resistant OCD focused on the SMA (Lee *et al.*, 2017; Arumugham *et al.*, 2018; Singh *et al.*, 2019; Pelissolo *et al.*, 2016; Talaei *et al.*, 2009; Hegde *et al.*, 2016; Mantovani *et al.*, 2010). Results suggest that 1 Hz rTMS over the SMA could be an efficient and safe add-on therapeutic method in treatment-resistant patients with OCD.

### Treatment modality and stimulation frequencies

In regard to differences in low and HFs of rTMS, results from the extracted studies suggest that, administration of HF (10 Hz) rTMS at either 100% or 110% MT over the RDLPFC did not differ from sham rTMS in terms of efficacy in relieving symptoms, reducing clinical severity or improving responses in treatment-resistant OCD (Mansur *et al.*, 2011; Elbeh *et al.*, 2016). By contrast, another study indicated that low frequency (1 HZ) rTMS delivered to the RDLPFC appeared to be superior to sham rTMS for relieving OCD symptoms and depression, in patients with treatment-resistant OCD. Based on the results from the selected studies in this review, there is no evidence for a statistically significant difference between low or HF rTMS over RDLPFC and LDLPFC for the treatment of OCD.

The different study designs did not contribute to any differences in the outcomes for treatment between the sham and active subjects. A study conducted (Sachdev *et al.*, 2007; Praško *et al.*, 2006) using the double-blind, randomized, sham-controlled trial with the application of low or HF rTMS over the left or right PFC presented with a significant reduction in YBOCS scores in both sham and active subjects with no significant statistical difference in the two groups at the end of the treatment intervention. The results also failed to depict any meaningful therapeutic efficacy in treatment non-responder OCD patients from either of the groups (Kang *et al.*, 2009).

Sachdev *et al.* (2001) compared effects of active HF-RDLPFC rTMS to active HF-LDLPFC rTMS. The evaluation yielded notable improvement in the symptoms of the OCD in study subjects. Notwithstanding the significant improvement in YBOCS scores for the two arms of the study, it is possible that the positive results were because of the smaller sample size (*N* = 12) and also the absence of a control group. These same researchers six years later conducted a similar study that confirmed the assertion of a smaller sample size and the lack of a sham control. Sachdev *et al.* (2007) in their study with a larger sample size (*N* = 18) revealed that the active and sham arms of the study did not show any difference in the reduction in OCD symptoms after the treatment. These conflicting results indicate that prefrontal high or low frequency rTMS may probably not be effective in the treatment of OCD symptoms.

In contrast to the contradictory results from other studies, most of the trials that presented with major clinically insignificant improvements in OCD symptoms were the studies with the targeted brain regions over the SMA with low frequency rTMS (Donse *et al.*, 2017; Lee *et al.*, 2017; Arumugham *et al.*, 2018; Singh *et al.*, 2019; Pelissolo *et al.*, 2016; Talaei *et al.*, 2009; Hegde *et al.*, 2016; Mantovani *et al.*, 2010; Gomes *et al.*, 2012; Rostami *et al.*, 2020) and also the left OFC with LF-rTMS (Kumar *et al.*, 2018; Singh *et al.*, 2019; Ruffini *et al.*, 2009). These studies suggest that rTMS had a specific and significant clinically effective influence on OCD symptoms: specifically in relation to the SMA stimulation site.

Poor study outcomes as witnessed in most of the studies could be partly attributed to differences in stimulation parameters, shorter treatment durations (as many used two weeks), the levels of frequencies used and, in some cases, the use of the circular coil, which typically induces less focal current compared to the figure-of-eight shape coil. Differences may also be attributed to the choice of whether left or right prefrontal cortices of targets for stimulations and the severity of the drug resistance of the subjects used for the purposes of the studies.

### Other factors affecting therapeutic outcomes

Many factors may have accounted for the varied effectiveness of the application of rTMS across the studies and major domains of outcomes. For instance, rTMS treatment protocols and stimulation parameters vary greatly across studies, with poorly defined intervention protocols. Another factor is the different measurement tools used for the evaluation of similar outcomes across studies, and therefore, making comparison and evaluation of results difficult. These inconsistencies also make it difficult to understand which rTMS parameters lead to the most significant outcomes and treatment responses. It remains possible that positive outcomes may also be attributed partially to the therapeutic contributions of concurrent medications taken by the subjects although most of the subjects have been on these medications for a long time without yielding improvements in their OCD symptoms.

Additionally, the varied clinical significance and effectiveness of rTMS across studies can also be partly attributed to factors such as, variations in coil type and coil positions, the different cortical targets and the variations in motor thresholds. In the case of the application of rTMS for the treatment of OCD, a majority of the studies applied rTMS to normalize frontal dysfunction associated with OCD symptoms, choosing to stimulate the left/right DLPFC or the SMA. For example, in the case of the cortical target, the SMA was consistently used to relieve subjects of their OCD symptoms with consistent and clinically significant treatment responses noted. Thus, from the data gathered with respect to rTMS in OCD, it seems that the SMA may be a promising target region for the application of rTMS to treat the symptoms of OCD in contrast to either left or right DLPFC.

Furthermore, an important factor noticed is the evaluation of the longevity and time course effects of rTMS. The majority of studies reviewed evaluated the treatment outcomes of the various interventions immediately after the last session of rTMS with a few months of follow-up. Considering the chronic, debilitating and high prevalent nature of mental conditions, evaluating the long-term therapeutic effects of rTMS intervention is of great importance. Therefore, it would be of high clinical significance and research value to estimate the sustainability of treatment effects, and specifically, maintenance strategies following response or remission with rTMS.

### Limitations

The authors of this scoping review acknowledge several limitations. First, our search strategy considered only studies published in English and the results are up to date as the last day of the electronic data search of December 10, 2020. Though every effort was made to identify all relevant studies for the purposes of this review per our eligibility criteria, we still may have missed some relevant studies, especially those published in other languages.

## Conclusion

Many of the studies included in this scoping review presented with conflicting and inconsistent outcomes on the efficacy and utilization of rTMS as a treatment intervention for OCD. This makes it difficult to make definitive conclusions on the clinical usefulness and the appropriate technique for rTMS treatment interventions for OCD. Larger sample sizes for sufficiently powered and preferably multi-centered sham-controlled trials with the appropriate coil and stimulation parameters, well-defined stimulation targets and a longer treatment duration would be required to bring clarity to the therapeutic effect of rTMS in the treatment of resistant OCD.

Despite the inconsistencies in the published literature, the application of rTMS over the SMA and the OFC has proven to be promising in efficacy and tolerability compared with other target regions such as PFC for the treatment of OCD. Despite the diversity in terms of the outcomes and clinical variability of the studies under review, rTMS appears to be a promising treatment intervention for OCD.

## Figures and Tables

**Table 1:** Medline search strategy

**#**	Search strategy	Results
**1**	exp *stress disorders, post-traumatic/ or (PTSD or ((posttraumatic or post traumatic or combat or war or trauma*) adj1 (stress* or neurosis or neuroses or nightmare*)) or ((traumatic or acute) adj (stress disorder* or stress symptom*)) or shell shock* or shellshock*).mp	**46,596**
**2**	exp obsessive-compulsive disorder/ or bipolar disorder/	**54,776**
**3**	(Bipolar or bi-polar or manic-depress* or mania or obsessive-compulsive disorder* or OCD).mp	**102,961**
**4**	1 or 2 or 3	**147,991**
**5**	Transcranial magnetic stimulation/	**11,653**
**6**	(repetitive transcranial magnetic stimulation or rTMS).mp	**5,423**
**7**	5 or 6	**13,372**
**8**	4 and 7	**492**

**Table 2:** Summary of studies using rTMS for the treatment of OCD

**Author (year)**	Country of origin	Study design	No. of participants	Targeted brain region	Targeted symptom	Measurement	Duration of treatment	Coil/ rTMS parameters/stimulation method	Outcome/significant improvements	Assessment and follow-up	Conclusion	Side effects
**Sachdev *et al.* (2007)**	Australia	Double-blind, randomized, sham controlled followed by open-label phase	18 adults	Left DLPFC	Obsessive symptoms	YBOCS MADRS BDI STAI-I	TWO weeks	Focal 8-shaped 70 mm coil, with 30 trains of 5 s each, at 10 Hz and 110% MT, with 25-s inter-train intervals (1,500 stimuli per session)	This study did not support the efficacy of high frequency Left DLPFC rTMS given over two weeks In OCD, as there was no improvement in obsession scores	Weekly throughout the study and after one and six months of the last treatment	Two weeks of rTMS over the left DLPFC is ineffective for treatment-resistant OCD	Transient headache, localized scalp pain
**Kang *et al.* (2009)**	Republic of Korea	A double-blind sham-controlled investigation	21 patients	Right DLPFC	Effect of rTMS on cognitive functions Anxiety symptoms Obsessive compulsive symptoms	YBOCS MADRS	10 days	Focal 8-shaped 70 mm coil, with daily sessions for the first 2 weeks At 1 Hz and (100% and 110%) RMT, at 10 min (1,200 stimuli/d)	The study did not show any clinically meaningful efficacy of sequentially applied low-frequency rTMS over a right DLPFC and SMA of patients with OCD	At baseline, after one and two weeks of stimulation and two weeks after the final session	The study did not show any clinically meaningful efficacy of sequentially applied low-frequency rTMS over a right DLPFC and SMA of patients with OCD	Transient headache, localized scalp pain
**Mantovani *et al.* (2010)**	USA	This trial consisted of two phases, namely, 4-wk double blind and 4-wk open-label	21 Patients with 8 women	Coil was positioned over pre-SMA	Increases in right hemisphere MT and normalization of baseline hemispheric asymmetry of cortical excitability	HAMD 24, YBOCS CGI-S, BDI-II HAMA-14	4-wk double blind and 4-wk open-label	A vacuum cooled 70-mm figure-of-eight coil. Stimulation of 1-Hz, 20-min train at 100% MT, once a day, 5 d/wk., for 4 wk. (in Phase 1) to 8 wk. (in Phase 2	There was an average of 25% reduction in the YBOCS compared to a 12% reduction in those receiving sham. For the 4 wk. and for the 8 wks. 28.2 + −5.8 to 14.5+ −3.6	Every two weeks and self-rating forms filled at the end of every week	There was an average of 25% reduction in the YBOCS compared to a 12% reduction in those receiving sham. For the 4 wks. and for the 8 wks. 28.2 + −5.8 to 14.5 + −3.6	nil
**Sachdev *et al.* (2001)**	Australia	Single-blind, randomized, non sham controlled	12 patients	Right DLPFC And L DLPFC	To compare the efficacy of both RDLPFC and LDLPFC	YBOCS, MADRS, BDI, STAI-I	Two weeks	Active RDLPF**C**10 sessions, RDLPFC, 10 Hz, 110% MT, 15 min, 30 trains, 5 s on, 25 s off, fi g-8 coil Active LDLPFC idem, LDLPFC	Global reduction in YBOCS score _ 40% from baseline to wk. 2 and wk. 6	At baseline, two weeks, six weeks	Significant improvement in relieving OC symptoms, reducing clinical severity or improving treatment response; for both LDLPFC and LDLPFC	Nil
**Gomes *et al.* (2012)**	Brazil	Randomized double-blind trial	22 right-handed outpatients (women: 13; men: 9), age 18 to 60 years	Coil positioned over pre-SMA	To assess the efficacy of low-frequency rTMS to the SMA in treatment-resistant OCD and further examine the duration of a significant clinical effect	HAMD YBOCS	Two weeks	Focal 8-shaped,70-mm coil with 1-Hz, 20-min trains (1,200 pulses/day) at 100% MT. once per day, five days per week, for two weeks	No significant reduction in Y-BOCS for baseline but at 2 wks, there was a significant reduction for the active group. No significant difference between groups for anxiety and depression symptoms	baseline, after rTMS treatment and 14 weeks after the end of rTMS treatment	No significant reduction in Y-BOCS for baseline but at 2 wks, there was a significant reduction for the active group. No significant difference between groups for anxiety and depression symptoms	Mild headache, scalp discomfort, cervical pain
**Ma *et al.* (2014)**	China	Double blind sham-controlled study	46 patients completed after 2 treatments9 inpatients and 37 outpatients. Aged between 18 and 60	Bilateral DLPFC	Obsessive, depressive and anxiety symptoms in OCD patients	HAMD YBOCS HRSD, CGI	Two weeks	A 9 cm circular coil. 80% MT. Daily for 5 sessions a wk. for 2 wks. with 20 min. each min included 4 s of active stimulation and 56 s of rest	The result showed that there were changes in scores of YBOCS, HRSD and HAMA over time following both α-TMS and sham treatments	Baseline, after the 5th and 10th sessions of treatment and 1 wk. after completing the entire treatment	αEEG-guided TMS may be an effective treatment for OCD and related anxiety	Mild headache
**Nauczyciel *et al.* (2014)**	France	A randomized, double-blind, crossover design	19 patients	Right orbitofrontal cortex (OFC)	Reduction in clinical symptoms, as measured on the Y-BOCS	YBOCS MADRS CGI	Two per day for one week	DB-80 butterfly double-cone coil with 120% MT, 1 Hz, 1,200 pulses per session over the right OFC. 10 sessions, two per day over one week	At day 7, a significant decrease in Y-BOCS scores, was observed compared with baseline, at day 35, no difference was observed in this decrease from the Y-BOCS baseline between active and sham stimulations	Assessments were performed before and after each sequence, as well as one month after the end of the last session	Results of this preliminary study suggest that the OFC is a possible neuroanatomical target for OCD treatment, especially rTMS	Nil
**Donse *et al.* (2017)**	The Netherlands	An open-label design	22 patients	Bilateral SMA and right dorsolateral prefrontal cortex (DLPFC)	Role of sleep disturbances in OCD and its predictive value for rTMS treatment nonresponse	Y-BOCS, BDI, PSQI	10 sessions	Using a figure-eight-coil with a frequency of 1 Hz, 1,000 pulses per session, 110% MT.10 sessions over the SMA	Study confirms that some sleep disturbances are more prevalent in OCD patients than healthy subjects	Baseline and after the 10 sessions	Findings suggest that CRSD variables can predict treatment non-response to rTMS in a sample of treatment-resistant OCD patients	Nil
**Lee *et al.* (2017)**	Republic of Korea	An open–label pilot study	9 adults aged 18 or older	SMA	Obsession and compulsion symptoms of OCD	BAI Y-BOCS, BDI CGI-GI SCL-90-R	Five days a week for four weeks	70 mm, 8 shaped coils.1 Hz, 20 min train (1,200 stimuli/day) at 90–100% RMT, once a day, 5 days a week, for 4 weeks, in 20 sessions	Symptoms in treatment-resistant OCD patients significantly decreased after 20 sessions of 1 Hz rTMS over the SMA	Baseline, after two weeks and after four weeks of rTMS treatment	Findings suggest that 1 Hz rTMS over the SMA can be an efficient and safe add-on therapeutic method in treatment-resistant patients with OCD	Mild headache and mild dizziness
**Kumar *et al.* (2018)**	India	A retrospective open study	25 patients	LF-rTMS over left-OFC	Symptoms of OCD, factors affecting response to rTMS	Y-BOCS	Four weeks	1-Hz at 110% TM 5-s train duration, intertrain interval of 10 s and 240 trains per session. 20 sessions 5 days per wk. for 4 wks	Significant reduction in the mean YBOCS scores after completion of 20 sessions of rTMS from baseline, whereas no further significant change in YBOCS scores one month after completion of rTMS treatment	Baseline and one month after the treatment	There is a role of applying LF-rTMS over Lt-OFC as an augmentation strategy in ameliorating clinical symptoms among patients with medication-refractory OCD	Localized scalp discomfort, headache
**Arumugham *et al.* (2018)**	India	A randomized controlled trial	40 patients with 36 patients in analysis-19 received active rTMS and 17 received sham	Low-frequency rTMS over pre-SMA	Reduction in clinical symptoms, as measured on the Y-BOCS	HAM-D YBOCS CGI-S HAM-A	Three weeks	Fluid cooled figure-of-eight coil (MCF-B70 butterfly coil. 1,200 stimuli per day at 1 Hz in 4 trains of 300 s, with intertrain interval of 2 min, at 100% MT	Low-frequency rTMS over pre-SMA was not superior to placebo in reducing symptoms of OCD in partial/poor responders to SSRIs	0, 1, 2, 3 and 12 weeks using YBOCS	Low-frequency rTMS over pre-SMA may not be effective as an augmenting agent in partial/poor responders to SRIs	Headache, sedation, concentration difficulties and failing memory
**Singh *et al.* (2019)**	India	Retrospective review and analysis of records	79 patients	Left-OFC and over bilateral SMA	Reduction in clinical symptoms, as measured on the Y-BOCS	YBOCS	Four weeks	70-mm figure of-eight air-film coil.1-Hz at 110% RMT, 5-s train duration, intertrain interval of 10 s and 240 trains per session. Each session consisted of 1,200 pulses/d delivered in 3,590 s. A total of 20 sessions of rTMS 5 days per week for 4 weeks	Significant reduction in the mean YBOCS score after 20 sessions of rTMS, as compared with baseline YBOCS score	First day before the beginning of rTMS session and after the completion of 20th rTMS session	This study provided evidence for overall effectiveness of adjunctive 1-Hz rTMS treatment over either SMA or OFC in patients with medication-refractory OCD	Nil
**Mansur *et al.* (2011)**	Brazil	Parallel, double-blind randomized trial	30 patients 18– 65 years	R-DLPFC	Scores on the YBOCS and CGI-I scale	HAM-D YBOCS CGI-S HAM-A CGI-I	Six weeks	Figure-of-eight coil 10 Hz and at 110% MT. 30 sessions (1/d, 5 d/wk.).40 trains – 5 s per train, with a 25-s intertrain interval. Total 60,000 pulses	rTMS, over rDLPFC, was not found to be superior to sham rTMS in relieving OC symptoms, reducing clinical severity or improving treatment response	Baseline; after 2 and 6 wk. treatment; and after 2 and 6 wk. follow-up	Active rTMS over the rDLPFC does not appear to be superior to sham rTMS in relieving OC symptoms, reducing clinical severity or improving treatment response	Mild headache, scalp discomfort, cervical pain, mood swings
**Rostami *et al.* (2020)**	Asia	Retrospective study	65 patients	DLPFC or SMA	Y-BOCS	Y-BOCS BDI-II CGI-I BAI	Three days per week for seven weeks	70-mm figure-of-eight-coil (air film coil). 120% of AMT 1 Hz, for 30 min, total of 1,800 pulses per session. once a day, 3 days per week for 7 weeks, in 20 sessions (36,000 pulses)	Significant reduction in OCD symptoms and anxiety/depressive states were observed after 20 sessions of rTMS	Baseline and after the 20th session of rTMS	An overall significant reduction in OCD symptoms and anxiety/depressive states were observed after 20 sessions of rTMS	Headache and dizziness
**Ruffini *et al.* (2009)**	Italy	A randomized controlled investigation	23 patients 18–75 years	Left OFC	OCD symptoms, mood and anxiety	YBOCS, HDRS, HARS	Five sessions per week for three weeks	70-mm 8-shaped coil.10 min 1 Hz left-sided subthreshold rTMS 80% MT. 15 sessions (1 per day, 5 per week for 3 weeks)	Significant improvement in OCD symptoms in OCD patients with benefits lasting up to 10 weeks after the end of rTMS treatment	Baseline, after 15 rTMS sessions and every 2 weeks for 3 months after the end of rTMS	Low-frequency rTMS of the left OFC produced significant but time-limited improvement in OCD patients compared to sham treatment	Nil
**Mantovani *et al.* (2006)**	USA	Open-label pilot study	10 right handed outpatients	SMA	YBOCS, CGI	YBOCS, YGTSS, CGI, HARS HDRS, SAD, BDI SCL-90	10 days	70-mm figure-of-eight coil, SMA for 10 daily sessions at 1 Hz, 100% MT, 1,200 stimuli/day	Significant improvement in OCD and TS symptoms with benefits lasting up to three months. Improvements in depression and anxiety were also seen	Baseline and after 1 and 2 wk. of stimulation and 1 and 3 months follow up on CGI	Slow rTMS to SMA resulted in a significant clinical improvement and a normalization of the right hemisphere hyperexcitability, thereby restoring hemispheric symmetry in motor threshold	Nil
**Praško *et al.* (2006)**	Czech Republic	A randomized, double blind, sham controlled study	33 right-handed patients	Left DPLFC	General psychopathology	CGI, HAMA, Y-BOCS BAI	Two weeks	Air cooled, figure-of-eight 70-mm coil.1 Hz at 110% MT. 10 sessions. 30 min (5 per week for 2 weeks. 1,800 pulses per session	Low frequency rTMS of left prefrontal cortex had no impact on the symptomatology in the patients suffering with SSRIs resistant OCD	Week 0, week 2 and week 4	Low frequency rTMS administered over the left DPLFC during 10 daily sessions did not differ from sham rTMS in facilitating the effect of serotonin reuptake inhibitors in OCD patients	Nil
**Elbeh *et al.* (2016)**	Egypt	Double blind randomized clinical trial	45 patients	Right DLPFC	Effects of 1 Hz and 10 Hz on scales	Y-BOCS, HAM-A, CGI-S	Two weeks	70 mm figure-of-eight coil 1 Hz-rTMS at 100% RMT, 4 trains, each of 500 pulses with a 40 s and 10 Hz rTMS at 100% RMT applied in 10 trains of 200 pulses, with 20 s. total of 2,000 pluses (5 days/week) 2 weeks	1 Hz rTMS over the right DLPFC has medium term effect on obsessive-compulsive symptoms and anxiety	Before and after the last treatment session and three months later	There was a significantly larger percentage change in GCI-S in the 1 Hz group versus either 10 Hz or sham. We conclude that 1 Hz-rTMS, targeting right DLPFC is a promising tool for treatment of OCD	Transient headache
**Pelissolo *et al.* (2016)**	France	Sham-controlled trial	40 patients	Pre-SMA	Efficacy of 1-Hz rTMS over pre-SMA	Y-BOCS, CGI-S	Four weeks	70-mm figure-of-eight coil.1 Hz, 26-min sessions (four 5-min trains interval of 2 min, 1,500 pulses/d), at 100% of RMT	Low-frequency rTMS delivered to pre-SMA during four weeks had no better effects on drug refractory OCD patients than sham stimulation	Baseline and four weeks and follow-up (week 12)	Low-frequency rTMS applied to the pre-SMA seems ineffective for the treatment of OCD patients at least in severe and drug-refractory cases such as those included in this study	Headache
**Seo *et al.* (2016)**	Korea	A randomized controlled trial	27 patients	Right DLPFC	OCD symptoms, mood and anxiety symptoms	YBOCS, CGI-S HAMD	Three weeks	TAMAS stimulator with a figure-eight coil.1 Hz, 20-min trains (1,200 pulses/ day) at 100% MT once per day 5 days per week. for 3 weeks	LF rTMS over the right DLPFC appeared to be superior to sham rTMS for relieving OCD symptoms and depression in patients with treatment-resistant OCD	Baseline and every week during the treatment period	LF rTMS over the right DLPFC appeared to be superior to sham rTMS for relieving OCD symptoms and depression in patients with treatment-resistant OCD	Localized scalp pain, headache
**Talaei *et al.* (2009)**	Iran	A case report	40-year-old female	SMA	OCD symptoms, mood and anxiety symptoms	Y-BOCS	10 sessions	10 sessions with 110%, 1 Hz and of 30 min per day (a total of 1,200 pulses per day	Significant decrease in compulsive behaviors	Before the first rTMS session and after every session	Significant decrease in compulsive behaviors and obsessive thoughts	Nil
**Badawy *et al.* (2010)**	Egypt	Randomized control trial	60 patients	LDLPFC	Mixed OCD symptoms and compulsive symptoms only	Y-BOCS	15 sessions	High frequency r-TMS (20 Hz).5 sessions per week for 3 weeks. high frequency r-TMS (20 Hz)	While r-TMS was not effective as a single treatment for OCD patients, it was effective as add-on treatment for OCD patients	Before the first r-TMS session and after completion of the 15 sessions	While r-TMS was not effective as a single treatment for OCD patients, it was effective as add-on treatment for OCD patients	Nil
**Elmedany *et al.* (2014)**	Egypt	Randomized control trial	20 patients (9 men and 1 female)	Left prefrontal area of the brain	OCD symptoms	Y-BOCS CGI	20 Hz 2 s for 20 min in 8 sessions every 48 h	Figure-of-eight or butterfly-shape coil. 5 cm forward and 2 cm to the below the center of the head. MT 90%; 20 Hz 2 s for 20 min in 8 sessions every 48 h	OCD patients have better response to *r* TMS for obsession symptoms more than compulsions especially those on pharmacological treatment	Before the first r-TMS session and after completion of the last	OCD patients after r-TMS has a better response especially those accompanied with pharmacological treatment	Nil
**Greenberg *et al.* (1997)**	USA	Brief report	12 patients	Right lateral prefrontal, a left lateral prefrontal and midoccipital site on separate days, randomized	Obsessive compulsive symptoms	Y-BOCS HARS	20 Hz/2 s per min for 20 min	Cadwell High Speed Magnetic Stimulator and a figure-eight-shaped coil. 80%MT, 20 Hz/2 s per min for 20 min	Results suggest that right prefrontal rTMS might affect prefrontal mechanisms involved in OCD	Baseline and post-stimulation	Results suggest that right prefrontal repetitive transcranial magnetic stimulation might affect prefrontal mechanisms involved in OCD	Nil
**Hegde *et al.* (2016)**	India	Retrospective analysis study	17 patients	Pre-SMA	OCD symptoms	Y-BOCS CGI-S	Three weeks	70-mm figure-of-eight coil 1-Hz at 100% MT over the pre-SMA 20 min, in 4 trains of 300 s (1,200 pulses per sitting	Only 1 patient met the criteria for response after one month of treatment initiation	Baseline and one month after initiation	Low-frequency rTMS over the pre-SMA may not be effective in treatment refractory OCD	Mild headache
**Carmi *et al.* (2019)**	Israel	Prospective multicenter randomized double-Blind placebo-controlled trial	100 patients	Dorsal mPFC	Safety, tolerability and efficacy of dTMS in OCD	YBOCS, CGI-S HAMD CGI-I	Six weeks	H-shaped coil design, 100% RMT. 20 Hz dTMS 2-s pulse trains and 20-s intertrain intervals, for a total of 50 trains and 2,000 pulses per session	Significant differences between the groups were maintained at follow-up	Baseline and I month follow up	High-frequency dTMS over the mPFC and anterior cingulate cortex significantly improved OCD symptoms and may be considered as a potential intervention for patients who do not respond adequately to pharmacological and psychological interventions	One patient had suicidal thoughts
**Haghighi *et al.* (2015)**	Iran	Randomized, single-blind, sham, controlled clinical trial with cross-over design	21 patients	L-DLPFC	OCD symptoms	Y-BOCS, CGI	Four weeks	70 mm double air film coil. 100% RMT at 20 Hz, in 750 total pulse. 25 min per cortex site, totaling 50 min for a session	Both self- and expert-reported symptom severity reduced in the rTMS condition as compared to the sham condition. Full- and partial responses were observed in the rTMS-condition, but not in the sham-condition	Baseline, after two and after four weeks of treatment	The pattern of results from this single-blind, sham- and cross-over design suggests that rTMS is a successful intervention for patients suffering from treatment-resistant OCD	Nil
**Modirrousta *et al.* (2015)**	Canada	Open-label study	10 patients	mPFC	Effect of low-frequency deep rTMS over the mPFC of patients with OCD	Y-BOCS	Two weeks	Double-cone coil at 110% RMT 1 Hz, 150 pulses (overall 1,200 pulses in one session) for 10 sessions	Significant reduction in OCD symptoms	Baseline, after 10 sessions same day as last rTMS treatment, 1 month after last session	Results suggest the use of low frequency deep rTMS as a promising and robust intervention in OCD symptom reduction	Electric shocking sensation and insomnia

Notes: MT = motor threshold, SMA = supplementary motor area Y-BOCS = Yale–Brown Obsessive-Compulsive Scale; Ham-D–24 = Hamilton Rating Scale for Depression–24-item; BDI–II, DLPFC = dorsal lateral prefrontal cortex, OFC = orbitofrontal cortex, RMT = resting motor threshold, CGI-I = clinical global impression. HAMA = Hamilton Anxiety Rating Scale, HRSD = Hamilton Rating Scale for Depression, YMRS = Young Mania Rating Scale, GAF = global assessment of functioning, MCCB = MATRICS Consensus Cognitive Battery. QIDS = quick inventory of depressive symptomatology, CAPS = clinician administered PTSD scale, BNCE = brief neurobehavioral cognitive examination, STAI = state trait anxiety inventory, SC-Q = self-administered comorbidity questionnaire, SCID = structured clinical interview for DSM-IV, IPF = inventory of psychosocial functioning, BRMAS = Bech-Rafaelsen mania scale, CRSD = circadian rhythm sleep disorder, SCL-90-R = Symptom Checklist-90-Revised, mPFC = medial prefrontal cortex
